# Synthetic Strategies of Supported Pd-Based Bimetallic Catalysts for Selective Semi-Hydrogenation of Acetylene: A Review and Perspectives

**DOI:** 10.3390/molecules28062572

**Published:** 2023-03-12

**Authors:** Xinxiang Cao, Ben W.-L. Jang, Jiaxue Hu, Lei Wang, Siqi Zhang

**Affiliations:** 1 Laboratory for Development & Application of Cold Plasma Technology, College of Chemistry and Chemical Engineering, Luoyang Normal University, Luoyang 471022, China; 2 Department of Chemistry, Texas A&M University-Commerce, Commerce, TX 75429-3011, USA

**Keywords:** palladium, bimetallic, catalysts, semi-hydrogenation, acetylene, ethylene, cold plasma

## Abstract

Selective semi-hydrogenation of acetylene is an extremely important reaction from both industrial and theoretical perspectives. Palladium, due to its unique chemical and physical properties, is the most active and currently irreplaceable metal for this reaction in industry, but the poor catalytic selectivity towards ethylene is also its inherent shortcoming. Introducing a secondary metal to tune a geometric and electronic structures of Pd nanoparticles and to create a synergistic effect is the most widely used strategy to effectively improve the overall catalytic performance of Pd-based catalysts. Thus, various supported Pd-based bimetallic catalysts for selective semi-hydrogenation of acetylene have been exploited in the past decade. Timely comparison, analysis, and summarizing of various preparation methods may offer a beneficial reference for the subsequent development of such catalysts. In this context, herein, the advances in synthesis strategies of catalysts, including nano-catalysts, single atom alloys (SAAs), as well as bimetallic dual atom catalysts are summarized systematically. Their advantages and disadvantages are comparatively discussed. Finally, future perspectives for the synthetic strategies of supported Pd-based bimetallic catalysts for selective semi-hydrogenation of acetylene are proposed.

## 1. Introduction

Ethylene is one of the most important and largest bulk chemicals in volume in the chemical industry, which is mainly used in the large-scale production of polyethylene (PE), polyvinyl chloride (PVC), ethylene oxide/ethylene glycol(EO/EG), ethylbenzene, ethanol, vinyl acetate, acetaldehyde and other chemical substances [1,2]. Therefore, the global production volume of ethylene production has reached more than 210 Mt in 2021 alone, which is expected to grow even further at a rate of more than 3% with the increasing demand for the downstream derivatives of ethylene [2,3]. Most of ethylene is currently obtained by cracking petroleum hydrocarbons (light hydrocarbons from natural gas processing plants, such as ethane, as well as processed products and secondary processed oil from refineries, such as naphtha and hydrocracking gasoline) [1,4]. However, using this process route, a trace amount of acetylene is inevitably produced as a by-product. Unfortunately, the acetylene impurity (usually in the range of 0.1~2 vol.%) contained in the ethylene raw material does great harm during the production of the downstream derivatives of ethylene [1,4,5,6,7,8]. The trace amount of C_2_H_2_, easily and irreversibly deactivates the Ziegler–Natta catalysts of ethylene polymerization, which sharply degrades the eventual quality of the ethylene-derived polymers. In addition, it also easily forms explosive oligomers, which brings safety risks to the downstream reaction systems [1,2,3,4,5,6,7,8]. In this context, the acetylene concentration is required to be removed to less than 5 ppm to meet the downstream production demand [1,4,5,8]. Acetylene impurities could be removed by several methods, among which the catalytically selective semi-hydrogenation to ethylene is the most widely used way in the industry due to its good economy, environmental friendliness, and convenience [5,6,9]. On the other hand, in academia, acetylene hydrogenation has also attracted much attention from researchers because it is usually selected to serve as a model reaction of selective hydrogenation to evaluate the performance and catalytic mechanism of catalysts [10].

**Figure 1:**
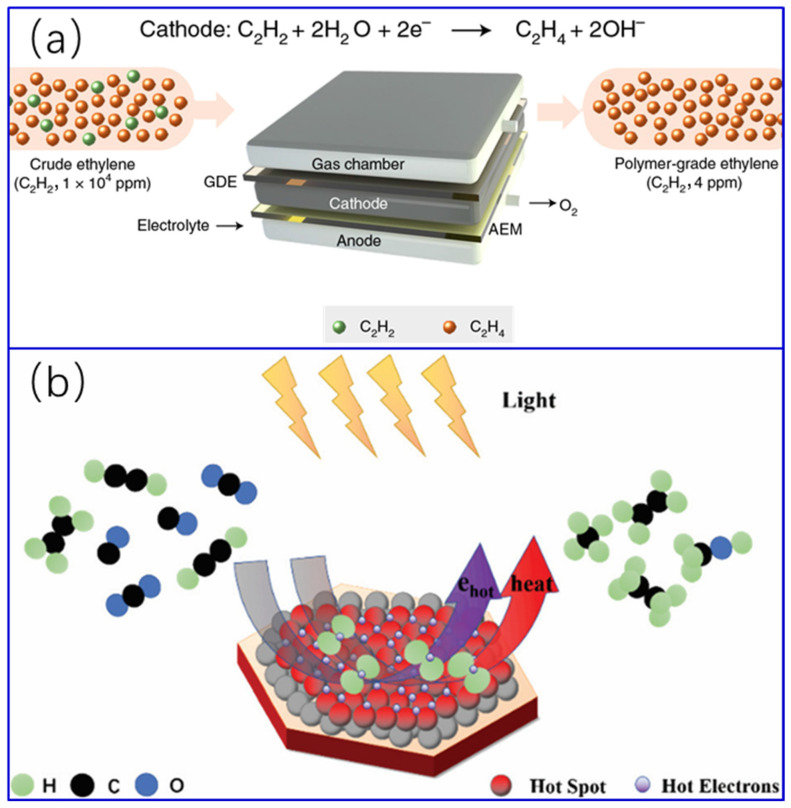
Schematic illustrations for the processes of (**a**) the electrocatalytic hydrogenation of acetylene proposed by Bu et al. [8] (Figure reproduced with permission from Ref. [8]. Copyright 2021, Nature Publishing Group) and (**b**) photothermal hydrogenation (Figure reproduced with permission from Ref. [11]. Copyright 2021, Royal Society of Chemistry).

At present, there are four processes for the selective catalytic semi-hydrogenation of acetylene, namely gas-phase, liquid-phase, electrocatalytic, and photothermal [11,12] hydrogenation of acetylene [1,8,13,14]. Gas-phase catalytic hydrogenation has been the focus of scientists and industrial engineers because the vast majority of ethylene purification plants are using this strategy [15,16]. It is typically carried out in multi-bed adiabatic high-pressure reactors via two different scenarios depending on feed condition, namely front-end and tail-end configurations [17,18,19]. In the tail-end process, the lighter components, such as CH_4_, CO, as well as excess hydrogen, are removed from the feed before entering the reaction systems. Therefore, for this process, the hydrogen–acetylene ratio can be artificially controlled to prevent over-hydrogenation of acetylene to form ethane. In contrast, in the front-end scheme, the feed steam directly enters the reactor without the removal of the light components in advance, and hydrogenation takes place with excess hydrogen, which tends to bring about over-hydrogenation of acetylene. Nevertheless, the front-end process also has its own advantages, that is, low green oil production, simple process, and low energy consumption. In consideration of the strong exothermicity of the gas-phase catalytic hydrogenation of acetylene, liquid-phase catalytic hydrogenation is definitely a safe option, which was firstly reported by Edvinsson et al. [20] in 1995. Simply speaking, for the liquid-phase hydrogenation process, the key equipment is typically a semi-batch reactor with a certain amount of pre-added solvent and catalyst; the feed steam continuously passes through this system at a given flow rate, and reaction occurs in the liquid [21,22,23,24,25]. In comparison with gas-phase catalytic hydrogenation of acetylene, solvent involvement in liquid-phase hydrogenation makes the heat transfer better; moreover, by selecting a suitable solvent, better catalyst stability and/or selectivity to ethylene can also be obtained [1,14,20]. In 1964, the feasibility of electrocatalytic semi-hydrogenation of acetylene was demonstrated by Burke et al. [26] using a palladized palladium electrode in an alkaline aqueous solution for the first time. Electrocatalytic semi-hydrogenation of acetylene can be carried out under ambient temperature and pressure. Especially the water in the electrolyte serves as a hydrogen source, avoiding the involvement of excessive hydrogen gas, which makes the process safer. The key component for such a reaction is the cell. Flow-cells are the most used devices at present [8,13,27,28]. Figure 1a is a schematic diagram of a process of electrocatalytic semi-hydrogenation of acetylene proposed by Bu et al. [8]. Although liquid-phase and electrocatalytic semi-hydrogenation of acetylene to ethylene have drawn ever-growing attention in recent years due to many merits compared with gas-phase catalytic hydrogenation; however, from the point of view of large-scale production, the technology is not mature enough, and there is still plenty of room for further improvement [1,8,13,14,20,26,27,28]. As the most ideal clean energy on earth, solar energy has become the focus of research, and especially the utilization of solar energy based on photothermal effects has become a very active research field in recent years. Photothermal catalysis is a new branch of catalytic chemistry, involving the integration of thermo- and photocatalytic processes, which can significantly improve catalytic activity and modulate catalytic reaction pathways and selectivity [29]. A specific photothermal hydrogenation process is as shown in Figure 1b. Most recently, photothermal catalysis is emerging for the semi-hydrogenation of acetylene [12]. The design of high efficiency photothermal catalysts with selective tunability to catalyze low carbon olefins is considered to be one of the research focuses in this field in the future [29].

**Figure 2:**
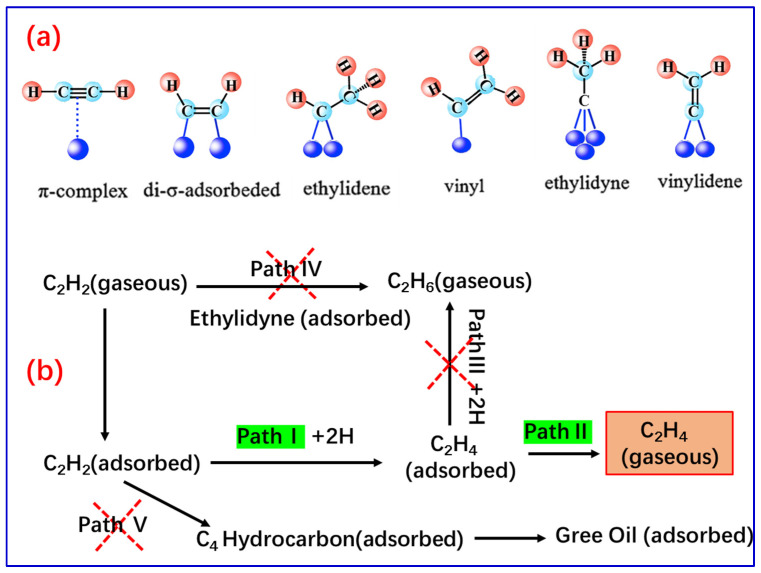
(**a**) Adsorption modes of acetylene on the Pd surface and [5,51] (**b**) possible reaction paths of acetylene hydrogenation [46,47].

There is no doubt that, from the perspective of economy and accessibility, the research and development of non-precious metal catalysts is an inevitable trend. At present, indeed, there are several non-precious metal catalysts emerging and showing potential for the selective semi-hydrogenation of acetylene, such as Ni-based [30,31,32] and Cu-based [8,33,34,35,36,37] catalysts. Even so, supported forms of Pd-based catalysts remain the industrial catalyst of choice to date [38,39,40,41,42,43,44]. Because Pd is the most efficient metal for this reaction due to its unique electronic structures as well as its superior ability to dissociate dihydrogen and activate acetylene, e.g., supported Pd nanoparticles can easily catalyze the reaction to achieve complete conversion of acetylene even at room temperature and normal pressure [40,41,42]. However, for supported Pd nanoparticles, over which the undesired over-hydrogenation of acetylene to ethane and the dimerization of acetylene further polymerization into green oil also easily occur, this results in lower ethylene selectivity and the catalytic activity [38,43,44]. The published theoretical calculation [44,45] and experimental results [46,47,48,49,50] can reveal the reason for the performance deficiencies of supported Pd nanoparticles for the selective semi-hydrogenation of acetylene to some extent. Studt et al. [44,45] found through theoretical calculation that Pd metal surfaces have strong adsorption on both ethylene and acetylene; in addition, the activation barriers for the hydrogenation and desorption of ethylene are comparable and relatively low, which inevitably leads to easy over-hydrogenation of acetylene to ethylene. On the reaction mechanism side, six adsorbed acetylene species on the Pd surface have been detected by means of spectroscopic techniques [46,47,48,49,50], and are shown in Figure 2a. They are π-bonded C_2_H_2_, vinyl, and vinylidene adsorbed on Pd single atoms; di-σ-bonded C_2_H_2_ and ethylidene adsorbed on Pd-dimers, as well as ethylidyne adsorbed on Pd-trimers, respectively. Among them, ethylidyne adsorbed on Pd-trimers and ethylidene adsorbed on Pd-dimers are easy to be over-hydrogenate directly following Path IV (Figure 2b), forming ethane eventually.

Zhang et al. [52] and Larsson et al. [53] demonstrated that adsorbed species with unsaturated bonds tend to form dimers by coupling-hydrogenation or hydrogenation-coupling, further polymerization to green oil (Path V), under certain conditions, e.g., some specific catalyst systems, high H_2_ pressure, etc. Moreover, even the unsaturated adsorbed species follow Path I, the semi-hydrogenation of acetylene to adsorbed ethylene species, which could be further over-hydrogenate to ethane (Path III), because these adsorbed ethylene species also exist as ethylidyne and ethylidene forms [42,48].

In view of the above, adequately weakening the adsorption of ethylene, and/or raising the kinetic barrier in the hydrogenation of ethylene, as well as suppressing the coupling-hydrogenation and hydrogenation-coupling to achieve high ethylene selectivity and low production of green oil, should always be the first and foremost challenge for Pd-based catalysts, both in the industry and academia [41]. To that end, considerable efforts have already been invested to improve selectivity toward ethylene over Pd-based catalysts by different methods [2,4,15]. Among them, fabrication of bimetallic catalysts with a secondary metal is one of the most investigated methods because it possesses a wide range of choice for the assistant metal and a variety of well-acquired skills for their syntheses, providing a lot of possibilities for optimizing the catalytic performance by flexibly tuning the electronic and geometric structure of the active sites [54,55]. A prominent example is the bimetallic Ag–Pd/Al_2_O_3_ catalyst developed in 1957 by Frevel et al. [56]. It was shown that the addition of Ag could greatly improve the selectivity of Pd for the hydrogenation of acetylene to ethylene. Excellent performance makes this catalyst the first choice in the industry to date [40,57]. There are several informative reviews focused on selective semi-hydrogenation of alkynes [13,15,40,58,59,60]. However, they are rarely concentrated on the systematic summary of palladium-based bimetallic catalysts for selective hydrogenation of acetylene. In addition, the rapid emergence of new Pd-based bimetallic catalyst systems for the selective hydrogenation of acetylene warrants a broader assessment and discussion of recent developments of their preparation methodologies. In this regard, in the following sections, we thus focus on the advances in synthesis strategies of the Pd based bimetallic catalysts for the selective semi-hydrogenation of acetylene. including nano-catalysts, single atom alloys (SAAs), as well as bimetallic dual atom catalysts, are summarized systematically. Their advantages and disadvantages are comparatively discussed. Finally, the outlook is summarized. We hope that this collection will be a valuable reference source for the design and preparation of Pd-based bimetallic catalysts for the selective semi-hydrogenation of acetylene. The terms “bimetallic” and “alloy” in this review are used interchangeably without deliberate emphasis on whether the added foreign metal atoms form a true solid solution, core–shell structure, or islands, etc., with Pd atoms.

## 2. Synthesis of Supported Pd-Based Bimetallic Nano-Catalysts

Back in 1957, a patent on a series of catalysts containing palladium and group Ib metals for the selective hydrogenation of acetylene to ethylene was granted to Dow Chemical Co. Midland, MI, USA) [56]. It is shown that the addition of group Ib metals could effectively improve the selectivity to ethylene. Since then, the importance of bimetallic catalysts has been recognized. Typically, bimetallic catalysts can be classified into two categories, namely supported catalysts, and unsupported catalysts. The former is the most widely used catalyst in the production of bulk, fine chemicals, and fuels in industry, involving reactions such as hydrogenation, dehydrogenation, hydrotreating, oxidation, deNOx reactions, Fischer–Tropsch synthesis, ammonia synthesis, etc. [61,62] Supports of catalysts can provide a large specific surface area, enhance the stability of the supported metals via anchoring [63], modify the electronic structure of supported metals by strong metal–support interaction (SMSI) [64,65], as well as provide a synergistic catalysis effect with supported metals [66,67], etc. For the fabrication of high-performance Pd-based bimetallic catalysts, it is necessary to design suitable preparation methods based on the understanding of various preparation strategies. Recently, there are some excellent reviews that summarize the preparation methods of the supported Pd-based bimetallic nano-catalysts [10,38,54,55,62,68,69]. However, some new synthetic technologies and new catalysts have not yet been classified because they have just been reported. In this context, herein, commonly used, and new preparation methods of supported Pd-based bimetallic catalysts for the selective semi-hydrogenation of acetylene is summarized and compared.

### 2.1. Wet Chemistry Technique

#### 2.1.1. Incipient Wetness Impregnation (IWI) Method

**Figure 3:**
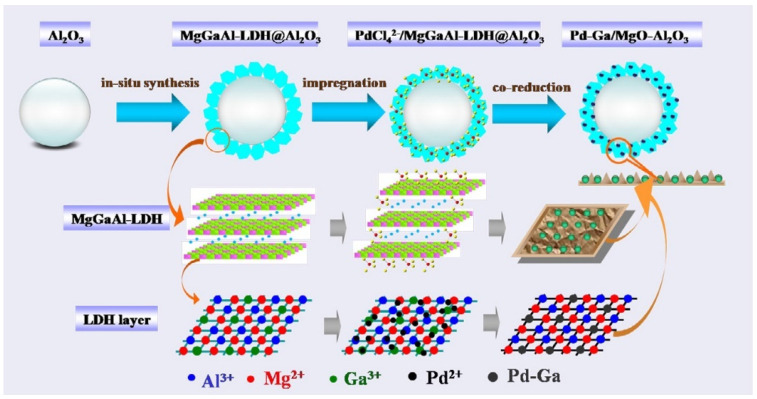
Synthetic schematic diagram of the novel supported bimetallic Pd–Ga catalysts. (Figure reproduced with permission from Ref. [72]. Copyright 2014, Elsevier B.V.).

The IWI method is a high-productivity, cost-effective, and straightforward approach that is widely used in the preparation of industrial Pd-based bimetallic catalysts for bulk chemicals, fine chemicals, and petrochemical products [68,69]. Depending on the impregnation sequence of two metal precursor solutions, (IWI) these methods are divided into two types: sequential incipient wetness impregnation (Seq-IWI) and incipient wetness co-impregnation (Co-IWI). The IWI method typically consists of four sequential steps [55]. First, the mixed solution of two metal precursors (for Co-IWI) is introduced onto a support, or two different metal solutions are successively impregnated onto a support. The intermediate nanomaterial will then be dried to remove water or solvent, followed by thermal calcination to form a catalyst precursor. Finally, bimetallic catalysts with catalytic activity would be prepared by thermal reduction of the precursors. However, the IWI method-derived Pd-based bimetallic catalysts normally govern large and un-uniform nanoparticles, resulting in low metal dispersion because of the weak interaction between metal precursors and catalytic support, even after the thermal treatment processes, i.e., calcination and thermal reduction [54,55,58]. Researchers have made a lot of efforts to solve these problems by optimizing the preparation process [70], supporting modification [68,71,72], as well as the introduction of emerging supports [71,72], etc. Our group [70] reported a modified sequential impregnation (MSI) method to prepare Al_2_O_3_ supported PdCu alloy catalysts for selective semi-hydrogenation of acetylene. Different from conventionally sequential impregnation (CSI), the MSI method adopted vacuum drying of precursors and eliminated the calcination step. It was found that for the catalysts prepared by the MSI method, Pd atoms formed a PdCu alloy on the surface of bimetal particles; however, for the catalysts prepared by the CSI method, Pd atoms tended to migrate and exist below the surface layer of Cu. Reaction results indicated that, compared to catalysts prepared with the former method, catalysts prepared by the MSI method possessed preferable stability with comparable reaction activity. In another case, Gu et al. [71] successfully obtained a supported palladium–gold alloy with an average particle size of only 2–8 nm by selecting the metal–organic frameworks (MOFs) with ethylenediamine (ED)-grafted MIL-101 as a support and using a simple liquid impregnation method. The ED-grafted MIL-101 MOFs possess a regular micropore structure and a large specific surface area, as well as the –NH_3_^+^ cationic groups in ED have a strong capture ability of PdCl_4_^2_^ and AuCl_4_^_^ ions, resulting in high dispersion of PdAu bimetallic nanoparticles after thermal reduction. In addition, He and coworkers [72] reported highly dispersed bimetallic Pd–Ga catalysts for the partial hydrogenation of acetylene by developing a new support. Initially, Mg–Ga–Al-layered double hydroxide (LDH) was synthesized in situ on spherical alumina to obtain a MgGaAl-LDH@Al_2_O_3_ precursor, followed by impregnation with PdCl_4_^2−^. They concluded that the electrostatic interaction between PdCl_4_^2−^ and positive charge of the MgGaAl-LDH layer enabled high dispersion of bimetallic Pd–Ga nanoalloys with the average size of ca. 2.6 nm. The synthetic schematic diagram is shown in Figure 3.

#### 2.1.2. Precipitation Method

The precipitation method is the other most efficient and simplest way of synthesizing supported Pd-based bimetallic catalysts [59,68,69,73]. Depending on the forms of support precursors (solid substrate or metal cation) introduced into the precipitation system, the precipitation method is divided into deposition–precipitation (DP) and co-precipitation (CP). Similar to the IWI method, the DP method typically consists of four steps: metal precursor loading on support, drying, calcination, and thermal reduction [68,69]. Nevertheless, the metal precursor loading on support for DP is carried out in the liquid phase. In more detail, the mixed solution of two metal precursors is introduced to a suspension of support nanoparticles. Next, with strong stirring, the pH of the liquid-phase mixture is adjusted by an acidic or basic reagent to make metal ion complexes deposit onto the support. The key objective of this method is to achieve simultaneous precipitation of different metals onto a support in suspension by controlling the processing conditions, including pH [73]. For CP, the difference from DP is that the support is also derived from its metal precursor by precipitation together with precursors of the other two target metals.

**Figure 4:**
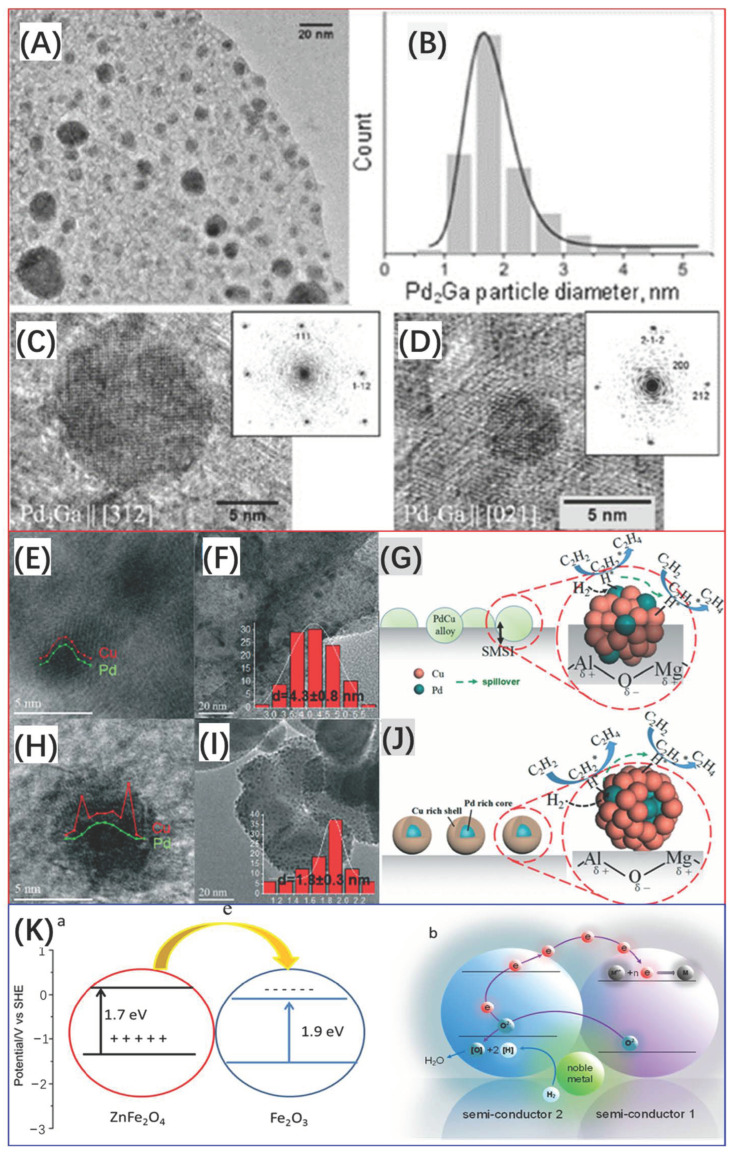
(**A**) HRTEM image of nano-Pd_2_Ga/MgO/MgGa_2_O_4_ with (**B**) particle size distribution and (**C**,**D**) corresponding FFT pattern after reduction at 773 K. (Figure reproduced with permission from Ref. [74]. Copyright 2014, ACS Publications). STEM–EDS line scanning and HRTEM images of the contrast sample, I-PdCu/MgAl-cHT (**E**,**F**) and Mco-PdCu/ MgAl-cHT (**H**,**I**) catalysts. Conceptual model for the selective hydrogenation mechanism of acetylene on (**G**) Mco-PdCu/ MgAl-cHT and (**J**) I-PdCu/MgAl-cHT catalysts. (Figure reproduced with permission from Ref. [75]. Copyright 2015, Royal Society of Chemistry). (**K**(a)) The exciton separation of ZnFe_2_O_4_–Fe_2_O_3_, a type II heterojunction. (**K**(b)) The proposed electron-flow cycle in the reduction process. (Figure reproduced with permission from Refs. [55,76]. Copyright 2015, John Wiley and Sons, Inc.).

Ota et al. [74] reported a series of Pd-substituted MgGa-hydrotalcite (HT)-like compounds prepared by the CP method with different Pd loadings for the selective hydrogenation of acetylene. Pd, Mg, Ga ternary hydrotalcite (HT)-like compounds (HTlc) precursors were synthesized by co-precipitation at 328 K and pH 8.5 by appropriate amounts of mixed aqueous metal nitrates (Pd^2+^ + Mg^2+^ + Ga^3+^ = 0.2 M) and 0.345 M NaCO_3_ solution as a precipitating agent. The nitrate solution was automatically pumped into a 2 L precipitation reactor (Mettler–Toledo LabMax) with a constant dosing rate, while the basic solution was added dropwise to maintain a constant pH of 8.5. After the precipitation, the liquid–solid mixture was aged for 1 h at 328 K. Subsequently, the solid was collected and dried, followed by decomposition in a reducing atmosphere at 773 K, a nano-Pd_2_Ga/MgO/MgGa_2_O_4_ catalyst was finally obtained. It is shown that the resulting sample exhibits monomodal and narrow particle size (2–6 nm) distribution (Figure 4A–D). A critical limit to avoid segregation is with ~1 mol % Pd due to the unique properties of HTlc and the thermal treatment under an H_2_ atmosphere. Liu and co-workers [75] reported a modified co-precipitation route to prepare MgAl-cHT-supported bimetallic PdCu catalysts containing only small amounts of Pd, Mco-PdCu/MgAl-cHT (Figure 4E–J). The characterization results show that the obtained PdCu catalyst contains uniform PdCu nanoalloys with an average size of only 1.8 ± 0.3 nm. As a comparison, bimetallic PdCu catalysts prepared by impregnation mainly contain a core–shell structure with an average size of 4.3 ± 0.8 nm. They demonstrated that the highly stable dispersed PdCu nanoparticles with small particle size and narrow distribution on the Mco-PdCu/MgAl-cHT catalyst are attributed to its high surface area as well as the net trap structure of MgO and Al_2_O_3_ crystals derived from the HT precursor. The Tsang group [55,76] designed a novel process to synthesize two-semiconductor oxides (ZnFe_2_O_4_–Fe_2_O_3_) supported by a PdFe bimetallic catalyst. Briefly, the CP method was employed to synthesize catalyst precursors with Pd(NO_3_)_2_, Fe(NO_3_)_3_⋅9 H_2_O, and Zn(NO_3_)_2_⋅6 H_2_O as metal and support precursors, followed by drying at 80 °C, and then by calcination at 300 °C for 2 h. Upon calcination, ZnFe_2_O_4_–Fe_2_O_3_ heterojunctions with staggered energy levels can be established. After H_2_ reduction at 250 °C, the final catalyst, PdFe/ZnFe_2_O_4_–Fe_2_O_3,_ was obtained. They demonstrated that due to the introduction of Zn ^II^ into the support, the reduction behavior of Fe ^III^ in H_2_ can be controlled rationally with the assistance of the formed Pd metal; as a result, supported PdFe bimetallic nanoparticles with controllable compositions and narrow particle size (1–2 nm) distribution can be obtained. The proposed scheme for the reduction mechanism of Fe ^III^ by the heterojunction is shown in Figure 4K. The excited electrons reside in the conduction band of Fe_2_O_3,_ and the holes reside in the valence band of ZnFe_2_O_4_. During the thermal reduction process, PdH_x_ species are formed and offer spilled atomic H to form water. Thus, corresponding electrons will reduce Fe ^III^ to Fe metal which further forms the PdFe alloy. This novel process may have the potential to be used to prepare other Pd-based bimetallic catalysts.

Compared with the IWI method, the CP method has a better control of particle size and distribution of bimetals even at high loadings; nevertheless, the operation process is relatively complex, and the operating conditions are stricter [59,68,69,73]. In fact, in most cases, the scattered particle size distribution is still the major issue faced by the precipitation method due to the subsequent thermal treatment process [68], which is albeit in favor of the formation of strong interactions between metal and support (SMSI).

#### 2.1.3. Sol-Gel Method

**Figure 5:**
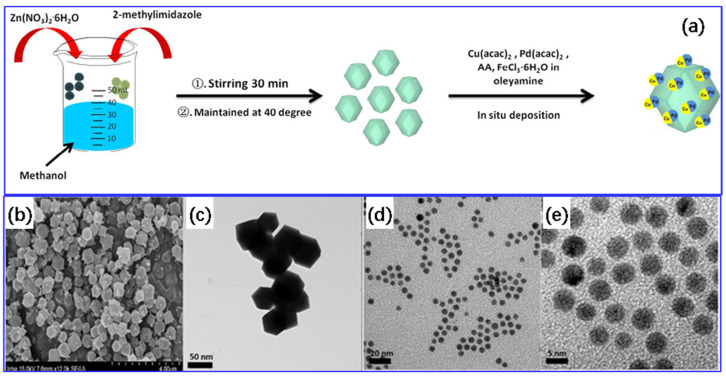
(**a**) Schematic illustration of the sol-gel procedure for the CuPd/ZIF-8 catalysts; (**b**) SEM and (**c**) TEM of ZIF-8; (**d**,**e**) HRTEM of CuPd bimetallic particles supported on ZIF-8. (Figure reproduced with permission from Ref. [79]. Copyright 2019, Elsevier B.V.).

Typically, the sol-gel method mainly consists of four steps. First, metal and support precursors are dissolved in a solvent to form a clear and stable colloidal solution (called sol) through primary condensation. Subsequently, the colloidal particles (micelles) undergo anisotropic condensation to produce polymeric chains, resulting in the formation of a transparent gel with a three-dimensional spatial network structure. After that, the gel is dried at low temperatures under reduced pressure, followed by thermal reduction to obtain the final sample [77,78]. Zhang et al. [79] employed the sol-gel method to synthesize ZIF-8-supported CuPd bimetallic alloy nanoparticles. To be more specific, firstly, 0.2 g of ZIF-8 NPs was added to 20 mL of oleylamine and sonicated for 1 h (solution A). Subsequently, 2.5 mg of Pd(acac)_2_, 2.2 mg of Cu(acac)_2_, 2 mg of FeCl_3_·6H_2_O, 5 mL of oleylamine, and 10 mg of ascorbic acid were mixed and ultrasonicated for 1 h (solution B). Solution A was quickly added to solution B. After stirring for 30 min, the mixture was heated to 160 °C and held for 5 h, followed by heating to 180 °C and holding for 3 h. The as-prepared catalysts were gathered, washed with a hexane/ethanol mixture (1:3), and finally dried at 30 °C in a vacuum oven for 12 h to obtain the targeted catalyst. The schematic illustration of the sample preparation is shown in Figure 5a. The ZIF-8 supported uniform CuPd spherical nanoparticles with an average particle size of 5.5 ± 1.0 nm were observed (Figure 5b–e).

In general, in the formation of gels, the reactants are likely to be mixed evenly at the molecular level, so that the sol-gel method can finally achieve a high degree of dispersion of metal active components on the support. Nevertheless, the raw material cost of this method is relatively high, and the use of organic solvents is not friendly to the environment and health.

#### 2.1.4. One-Pot Reduction Deposition (One-Pot RD) Method

The one-pot RD method can solve the issue of the scattered particle size distribution faced by precipitation methods to a certain extent because it normally avoids the thermal treatment processing. It is a one-pot method to prepare supported Pd-based bi-metallic catalysts in the liquid phase. Just as its name implies, this method is similar to deposition-precipitation. The difference between the two is that a reducing agent (e.g., hydrogen, ascorbic acid, hydrazine, borohydrides, and polyols) is introduced in the reduction-deposition system, and as a result, metal cations with different reduction potentials can be reduced by a one-pot liquid reaction to form supported bimetallic nanoparticles directly. Commonly, after further deposition onto a support as well as drying, the resulting sample does not need high-temperature treatment. Depending on the adding sequence of support and metal precures, the preparation process is slightly different.

**Figure 6:**
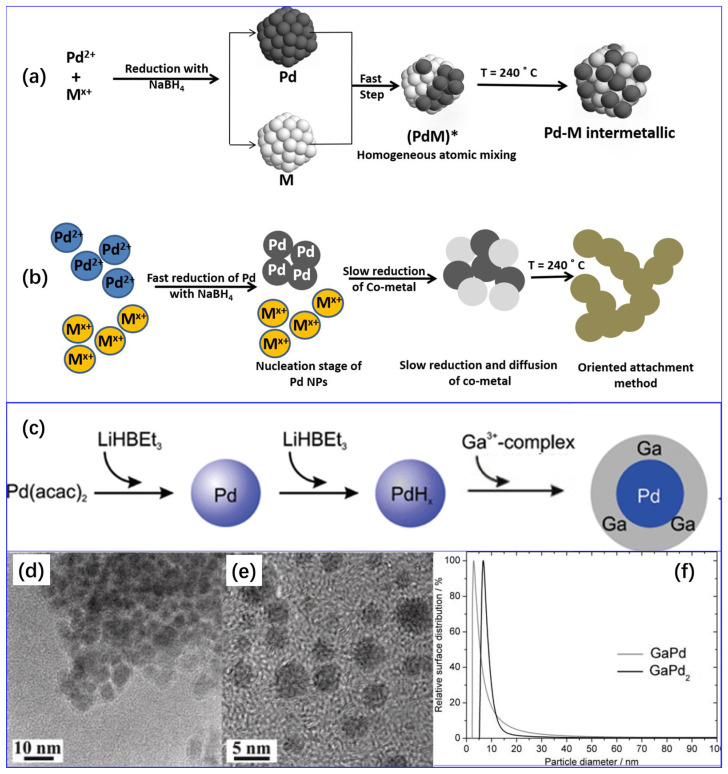
Schematic mechanism for the synthesis of Pd_x_M_y_ bimetallic compounds by the one-pot RD method in case of (**a**) small, and (**b**) big differences in reduction potential of Pd and metal M. (Figure reproduced with permission from Ref. [84]. Copyright 2019, American Chemical Society). (**c**) Proposed mechanism for the formation process of the Ga–Pd nanoparticle precursors; TEM images of the final Ga–Pd nanoparticles: (**d**) GaPd_2_ agglomerate; (**e**) GaPd nanoparticles; and (**f**) size distribution of GaPd_2_ and GaPd nanoparticles. (Figure reproduced with permission from Ref. [85]. Copyright 2011, ACS Publications).

Dodangeh et al. [80] employed this method to prepare mesoporous carbon nitride (MCN)-supported Ag-modified Pd catalysts for selective hydrogenation of acetylene. First, MCN and mono-ethylene glycol were mixed together, followed by ultrasonication for 4 h at 60 °C. After that, a desired amount of palladium nitrate and silver nitrate were added to the obtained solution and stirred for 12 h. The solution pH was adjusted to 11 by dropwise adding sodium hydroxide. Thereafter, the solution was refluxed at 90 °C under N_2_ protection for 6 h followed by filtration, washing, and drying at 80 °C in N_2_ atmosphere for 4 h. The characterization results show that the particle size of Pd nanoparticles is 2–8 nm. Lou and coworkers [81] developed highly selective acetylene semi-hydrogenation calcite-supported PdBi intermetallic compounds (PdBi/Calcite). Typically, 200 mg of calcite and 23.2 mg of B_i_(NO_3_)_3_·5H_2_O was dispersed in ethylene glycol aqueous solution (8 mL, 50 vol %) and stirred for 90 min. Subsequently, 2 mL of NaBH_4_ aqueous solution (5 M) was added ti the suspension during agitation in a dropwise manner. Next, 30 min later, an acetone solution with 4.24 mg of Pd(OAc)_2_ was added dropwise to the suspension above. After another 30 min, the precipitates were collected, washed with water and ethanol, and then dried in a vacuum oven at 40 °C. Before use, the catalysts were reduced in H_2_ at 200 °C for 1 h. The resulting PdBi/calcite catalyst showed good dispersion and a narrow particle size distribution (4.9 ± 0.7 nm). Yang et al. [82] reported a PdCu/C alloy catalyst for acetylene hydrogenation. A certain amount of Pd(acac)_2_, Cu(acac)_2_, and oleyl amine were mixed together and stirred for 1 h to form a solution, followed by heating at 200 °C under a nitrogen atmosphere for 12 h. The resulting PdCu nanoparticles were then centrifuged and washed with an ethanol/cyclohexane mixture to remove oleyl amine. Subsequently, the obtained PdCu nanoparticles were re-dispersed in cyclohexane during agitation, followed by the addition of 500 mg of carbon. After 2 h, the products were washed with ethanol and dried for 12 h at 60 °C. They found that a simple post-treatment at 375 °C could still maintain the average size of PdCu nanoparticles at 6.6–6.8 nm; however, dramatically improved ethylene selectivity at complete conversion of acetylene compared with untreated samples is due to the gradual replacement of Pd–Pd bonds by Pd–Cu bonds during the post-treatment process. Marakatti and coworkers [83,84] demonstrated that the difference between Pd and a secondary metal ion in reduction potential influenced the formation mechanism as well as the morphologies and structures of bimetallic nanoparticles in the one-pot RD process (Figure 6a,b).

Theoretically, if the potential difference is small, Pd (Pd^2+^/Pd, 0.915 V vs. NHE) and a secondary metal cation could be reduced almost simultaneously, and the reduced atoms could mix homogeneously and finally form uniform alloys. On the contrary, if the potential difference is large, e.g., the reduction potentials of Cu^2+^ (Cu^2+^/Cu, 0.34 V vs. NHE) is much lower than that of Pd^2+^, Pd^2+^ will be reduced first, and the secondary metal ions will be reduced on the surface of the formed Pd nanoparticles to further form the Pd@M core–shell (M, the secondary metal) structure. That is, if the reduction potential values of two metal cations are similar, catalysts tend to form uniform bimetallic alloys. However, for Ga, because its reduction potential (Ga^3+^/Ga, −0.53 V vs. NHE.) is negative and its strong Lewis acidity allows Ga^3+^-salts to form stable complexes with electron donors, it hinders the reduction of Ga^3+^. Moreover, the melting point of Ga is only 29.77 °C. As a result, the preparation of uniform Pd_x_Ga_y_ alloy is much more difficult to achieve in comparison with other Pd-based bimetallic alloys [85]. In this context, a modified reductive deposition method involving a two-step synthesis strategy for the preparation of Al_2_O_3--_supported single-phase GaPd and GaPd_2_ nanoparticulate by the co-reduction of metal precursors with LiHBEt_3_ in THF was developed by Armbr€uster et al. [85]. The formation of the nanoparticulate Ga–Pd precursors was confirmed to be due to the suitable solvent and reducing agent system, as well as the formation of Pd hydrides during the reduction process, resulting in the reduction and deposition of Ga on the surface of Pd particles (Figure 6c). TEM characterization results showed that the intermetallic GaPd and GaPd_2_ particles possessed unexpected narrow size distributions, and their mean particle sizes were only 3 and 7 nm, respectively (Figure 6d–f).

#### 2.1.5. Sequential Reduction-Deposition Method (S-RD)

S-RD is suitable for the deposition of a metal onto a ready-made supported parent metal in the liquid-phase environment to form bimetallic catalysts. Our group [86] as well as many other researchers [57,60,61] have demonstrated that a specific strong metal and support interaction (SMSI) could give a high catalytic performance for the selective semi-hydrogenation of acetylene. Noteworthy, in this method, the ready-made supported parent metal, which can be prepared by any suitable synthesis method, can also achieve SMSI. This approach can effectively adjust the loading amount of a secondary metal on to the parent metal, therefore demonstrating extraordinary potential in the synthesis of single atom alloys (SAAs) (detailed introduction of SAAs in Section 3. Synthesis of Single Atom Alloy and Dual Catalysts). Depending on the reduction mode of the secondary metal, the sequential reduction deposition method can be divided into electroless reduction deposition (eless-RD) [46,68,87,88,89], galvanic replacement (GR) [69,87,90,91,92,93,94,95], and controlled surface reactions (CSR) [55,61,87,96,97].

**Figure 7:**
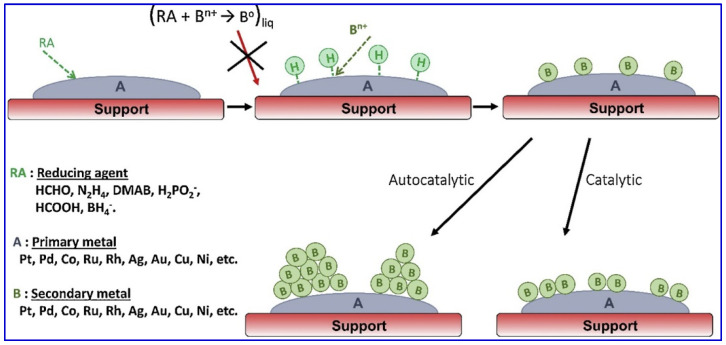
Schematic mechanism for electroless reduction deposition. Typically, metals A and B can be used interchangeably. (Figure reproduced with permission from Ref. [98]. Copyright 2018, Elsevier B.V.).

Eless-RD is also called electroless plating deposition. Just in terms of the synthesis procedure, eless-RD is basically the same as one-pot RD, except that the parent substrate is ready-made supported metal particles, as well as there is only one kind of metal precursor in the liquid phase for reduction and deposition. The reduction of the secondary metal in eless-RD refers to a class of reduction that occurs with the participation of chemical reducing agents, which do not involve electrochemical mechanisms. The mechanism of eless-RD is as shown in Figure 7 [98]. This scheme shows the activation of a suitable reducing agent on the parent metal surface, providing active sites, the metal hydrides, where the secondary metal cations are reduced and deposited as metal atoms. Pongthawornsakun et al. [99] reported bimetallic Cu–Pd/TiO_2_ prepared by eless-RD. In the method, Pd/TiO_2_ was used as a supported parent metal, and potassium dicyanocuprate (KCu(CN)_2_ was used as the Cu precursor for the synthesis of bimetallic Cu–Pd/TiO_2_ with hydrazine (N_2_H_4_, 35 wt% in H_2_O), and dimethylamineborane ((CH_3_)_2_NH·BH_3_; DMAB, 97%) as the reducing agent. The deposition of Cu was performed at 40 °C. The pH of the bath solution was maintained at 9.0 by the dropwise addition of NaOH solution. After 1 h of deposition, the slurry was filtered and the solid catalyst was washed with DI water, and then dried under vacuum at room temperature. In this case, Pd is used to activate the reducing agent, resulting in the chemisorption of H atoms on the Pd surface. These H atoms are identified as the active species to reduce the Cu precursor to Cu metal. Despite the successful deposition of secondary metal atoms on the surface of the supported parent metal nanoparticles achieved by using electroless deposition with precise adjustment of appropriate preparation parameters, how to ensure no deposition on the support is still a question to consider [46,68,87,100].

Galvanic replacement (GR) is involved in an electrochemical reaction between the supported parent metal and the other metal ions. The reduction potential difference between the parent metal and the other metal ions offers a driving force for the reaction [90,93,94]. For instance, Zhang et al. [101] employed the GR method to synthesize Pd–Ag/SiO_2_ bimetallic catalysts for the selective semi-hydrogenation of acetylene by replacing Ag from ready-made Ag/SiO_2_ nanoparticles with Pd. Briefly, a desired amount of palladium nitrate was completely dissolved in 100 mL 5% nitric acid, followed by the introduction of 1 g Ag/SiO_2_ nanoparticles into the solution. The galvanic replacement reaction occurred. The whole process lasted for 1 h and was carried out at room temperature, requiring intense agitation, and the dropwise addition nitric acid to maintain a pH of ~2. After the reaction, the filtered solid was washed thoroughly, followed by drying in vacuum oven at room temperature. The driving force of the reaction is the reduction potential difference between Pd (Pd^2+^/Pd is 0.915 V vs. NHE) and Ag (Ag^+^/Ag is 0.797 V vs. NHE). Their experiment results indicate that, as long as the amount of palladium in the solution is sufficient and the reaction time lasts long enough, the actual amount of palladium loaded onto Ag particles would be much larger than the amount of palladium required to cover the surface of all silver particles, in theory. This was attributed to the large difference in surface free energy between the loaded Pd and Ag, resulting in a two-way diffusion between the two metal atoms.

**Figure 8:**
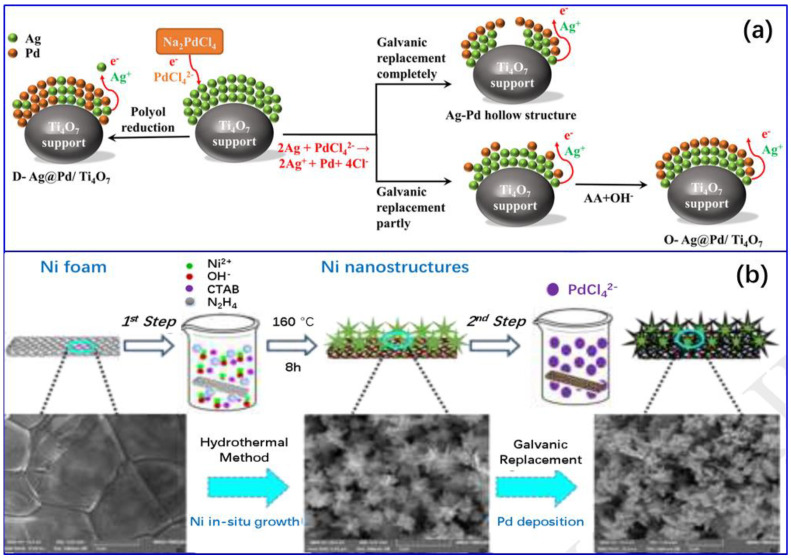
(**a**) The schematic illustration of the GR method and polyol reduction method synthesis for the Ag@Pd/Ti_4_O_7_ catalyst and (**b**) hierarchical Pd@Ni catalyst. (Figure reproduced with permission from Refs. [102,103]. Copyright 2018 and 2022, Elsevier B.V.).

By employing the GR method, various morphologies of bimetallic compounds can be constructed, such as core–shell [102], snow-like [103], etc. [104,105], as shown in Figure 8. Noteworthy, GR only occurs on parent metal nanoparticles; moreover, there is no addition of a reducing agent in the system; therefore, the deposition of the secondary metal onto the support can be effectively avoided by this method. However, this method is not appropriate for the deposition of metals whose reduction potential is lower than that of the supported parent metal. Regarding the Pd element, as a parent metal, it theoretically cannot be galvanically replaced by most other metals.

The controlled surface reaction (CSR) is similar to GR method in that both reactions occur on the surface of the parent metal. The difference is that the surface reaction for CSR is electroless. Therefore, CSR is particularly suitable for situations where supported Pd is the parent metal. For eless-RD, the unselectively physical adsorption or chemisorption of the metal precursors on the surface of a support is an issue that needs to be addressed [87,97,106], but with the CSR method, selective adsorption of the secondary metal precursors onto the parent metal can be achieved.

**Figure 9:**
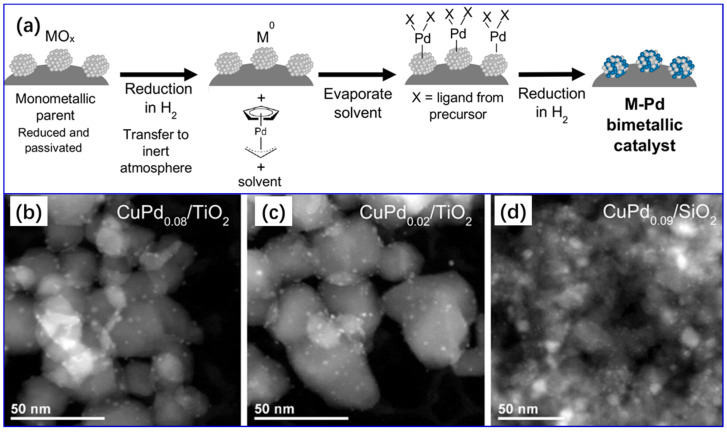
(**a**) Schematic of the CSR approach to synthesizing supported Pd-based bimetallic catalysts. The M is the parent metal. STEM images of catalysts with different Pd loadings: (**b**) CuPd_0.02_/TiO_2_, (**c**) CuPd_0.08_/TiO_2_, and (**d**) CuPd_0.09_/SiO_2_. (Figure reproduced with permission from Ref. [97]. Copyright 2020, ACS Publications).

Most recently, the Dumesic group [87,97,106,107] performed much meaningful work in this area. The typical procedure details for this method are shown in Figure 9. Surprisingly, the average particle sizes of the synthesized CuPd_0.02_/TiO_2_, CuPd_0.08_/TiO_2_, and CuPd_0.09_/SiO_2_ are extremely small, only 1.90 ± 0.51 nm, 1.63 ± 0.43 nm, and 2.81 ± 1.98 nm, respectively. The procedure is as follows: In a Schlenk tube, 1 g of parent catalyst was re-reduced under flowing H_2_ at 673 K. After cooling to room temperature under H_2_ atmosphere, the tube was sealed (Step 1). Next, a metal–organic complex containing (the secondary metal precursor) was completely dissolved in an organic solvent to form a solution. After that, the solution and the sealed Schlenk tube were moved to a glove box under a N_2_ atmosphere, followed by the solution being in contact with the parent catalyst after unsealing the Schlenk tube. The slurry was then stirred until the solution turned clear, suggesting a complete adsorption of the precursor from the solution onto the parent metal. FTIR and UV-vis spectroscopies were employed to evaluate the uptake of the precursor. The residual solvent was evaporated under an Ar atmosphere using Schlenk techniques (Step 2). The dried bimetallic catalyst precursor was then reduced in H_2_ at 773 K. Finally, the resulting bimetallic catalyst was then passivated with 1% O_2_ in He (Step 3). Steps 2 and 3 can be repeated to achieve higher metal loading, and the bimetallic catalysts were passivated under an O_2_ atmosphere only after the final cycle. With this method, highly dispersed bimetallic nanoparticles with uniform bimetallic nanoparticle size (~2 nm) and composition can be achieved.

#### 2.1.6. Photochemical Reduction (PR)

Most recently, the interest in the PR synthesis of metal nanocrystals of is growing rapidly due to its simplicity, high efficiency, as well as greenness resulting from generally not using any chemical or physical additives and being normally operated at room temperature and atmospheric pressure [68,69,108,109,110,111]. The unique feature of the PR method is the exposure of metal precursor-containing solutions to visible or ultraviolet (UV) light. Photogenerated electrons are used as reductants to reduce metal cations to metal atoms. However, the preparation of palladium-based bimetallic catalysts by photochemical reduction for selective hydrogenation of acetylene is rarely reported, which might be a direction of future efforts.

**Figure 10:**
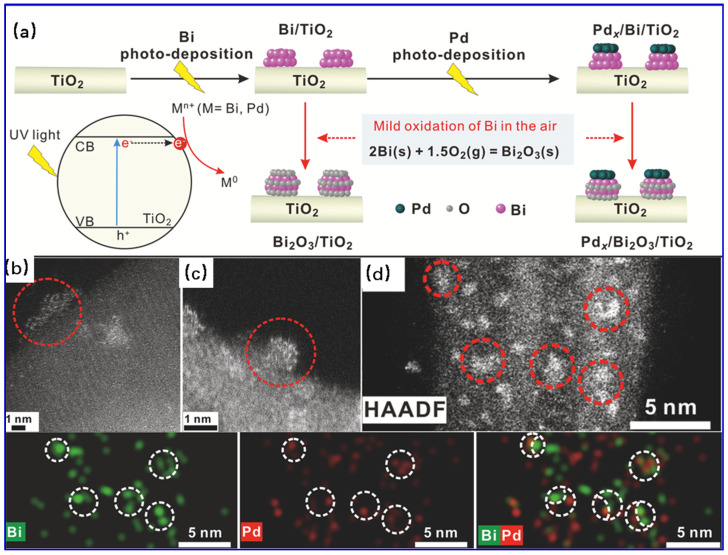
(**a**) Schematic illustration of the synthetic procedures of the PR method. STEM images of (**b**) Bi_2_O_3_/TiO_2_ with α-Bi_2_O_3_ clusters (highlighted by the red circle) and (**c**) Pd_1.0_/Bi_2_O_3_/TiO_2_ with Pd particles intergrown on Bi_2_O_3_ clusters (highlighted by the red circle). (**d**) Elemental mapping of Pd_1.0_/Bi_2_O_3_/TiO_2_. The clusters (highlighted by the white circles) are bicomponent with segregated Pd- and Bi-containing hemi-clusters. (Figure reproduced with permission from Ref. [109]. Copyright 2021, Nature Publishing Group).

The two most recent papers about catalyst preparation using the PR method are cited here to briefly describe the PR preparation process. Using a facile stepwise PR method, Zou and coworkers [109] fabricated a series of TiO_2_-supported hybrid Pd/Bi_2_O_3_ clusters for low-temperature acetylene semi-hydrogenation. A high pressure Xe lamp (300 W) is used as the light source in this experiment. Typically, 100 mg of TiO_2_ and 11.6 mg of Bi(NO_3_)_3_·5H_2_O were dispersed in 4 mL of ethylene glycol, followed by bubbling with Ar for 30 min to eliminate dissolved O_2_. Afterwards, the suspension was subjected to ultraviolet irradiation for 1 h. Subsequently, 8 mL of PdCl_2_ aqueous solution was introduced into the suspension followed by ultraviolet irradiation in an Ar atmosphere for another 1 h. The precipitates were collected, washed, and then dried in an oven at 40 °C. Prior to use, the obtained catalyst was activated by H_2_ at 100 °C for 1 h and then cooled to room temperature in N_2_. The schematically synthetic procedure as well as morphological and structural characterization results are illustrated in Figure 10. The demonstrated stepwise PR strategy in this work provides a new path for the fabrication of hybrid nanoclusters and nanometer metal/oxide interfaces. The Xiao group [112] employed the PR method to prepare a difunctional Zn–Ti layered double hydroxides (Zn–Ti LDHs) supported PdAu bimetallic catalyst for selective hydrogenation of phenylacetylene. In general, 0.1 g of Zn–Ti LDHs solid and 8 mL of deionized water were mixed and ultrasonicated, followed by the addition of 96 μL of HAuCl_4_ (9.7 mM) solution and 148 μL of PdCl_2_ (56.4 mM) solution. After agitation for 2 h under a N_2_ atmosphere in darkness, the PR treatments were performed on a Xenon-lamp parallel light source system (PLS-SXE300D/300DUV) for 30 min. The solid was collected, washed with deionized water, and finally dried in a vacuum oven to obtain the targeted Pd_9_Au_1_/ZnTi catalyst.

#### 2.1.7. Colloidal Synthesis

The boom in colloidal chemistry in the past two decades has made it possible to synthesize nanoparticles with uniform shapes, sizes, and compositions [89,113,114,115,116,117,118,119], because the metallic colloid is prepared in the presence of a chemical reducing agent (e.g., hydrogen, ascorbic acid, hydrazine, borohydrides, and polyols) and organic ligands, such as poly(vinylpyrrolidone) (PVP), cetyltrimethylammonium bromide (CTAB), poly(vinyl alcohol) (PVA), polyamidoamine (PAMAM), dodecyl mercaptan, trioctylphosphine, oil amines, oleic acid, etc. [38]. The former allows reduction to take place in the aqueous phase, so that the reduction is mild, fast, and controllable; the latter can serve as surfactants, capping ligands, and shape-directing agents to precisely control the directional growth of Pd-based bimetallic nanocrystals as well as prevent the coalescence of nanoparticles. Both the one-pot reduction deposition (colloidal one-pot RD) and the electroless reduction deposition (colloidal eless-RD), with the addition of organic ligands, are classified as colloid synthesis here [117,120].

**Figure 11:**
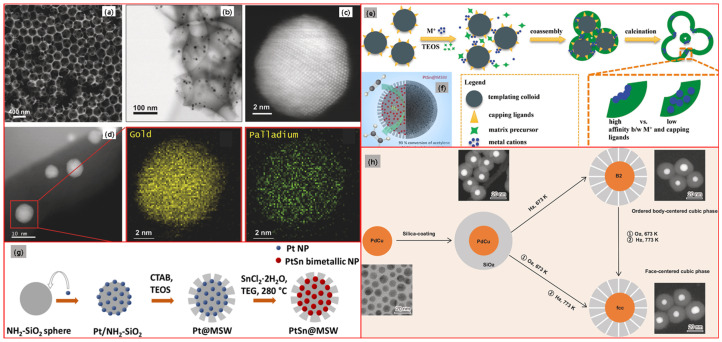
(**a**) SEM and (**b**,**c**) TEM images, as well as (**d**) the elemental mapping of Pd_0.04_Au_0.96_ RCT-SiO_2_ (Figure reproduced with permission from Ref. [116]. Copyright 2021, Nature Publishing Group) (**e**) Schematic of the formation of inverse opal (IOs) structures using proto-raspberry templating. (Figure reproduced with permission from Ref. [123]. Copyright 2017, John Wiley and Sons, Inc.). (**f**) TEM image and (**g**) schematic synthesis of Pt–Sn bimetallic nanoparticles encapsulated in a mesoporous silica well. (Figure reproduced with permission from Ref. [115]. Copyright 2020, John Wiley and Sons, Inc.). (**h**) Schematic illustration of the synthesis of silica–coated PdCu bimetallic catalysts with different crystal phases at the single-nanoparticle scale and TEM images of the representative structures of samples at different preparation stages. (Figure reproduced with permission from Ref. [114]. Copyright 2022, Nature Publishing Group).

With the colloidal synthesis method, PdCu bimetallic nanoparticles supported on carbon nanotubes (PdCu/CNTs) were developed by Lomelí-Rosales et al. [117] for the semi-hydrogenation of alkynes and acetylene. Under a nitrogen atmosphere, a certain amount of Pd(dba)_2_, CuMes, and CNTs were dispersed in 50 mL of dry THF in a Fisher Porter reactor, followed by the addition of Me_2_Im-CO_2_ (0.033 mmol). After that, the reactor was tightly closed, pressurized with three bars of H_2_, and subsequently kept at 70 °C and 700 rpm. Then, 16 h later, the mixture was cooled to room temperature. Next, the reactor was depressurized and moved into a glovebox with inert gas, and 20 mL of dry hexane was then introduced to the reactor followed, by stirring for 10 min. The average particle size of the obtained PdCu PdCu/CNTs is 2.8 ± 0.6 nm. Analogous to the deposition of metal ion precursors or small metal–organic molecule precursors, pre-synthesized colloidal bimetallic nanoparticles can also be loaded onto supports through adsorption [113,118,119,120,121,122], etc. The McCue group [119] produced core–shell structures (Au core and Pd shell) with colloidal synthesis to further synthesize TiO_2_ supported Au@Pd/TiO_2_ catalysts through adsorption. Briefly, Au nanoparticles were synthesized by the reduction of HAuCl_4_ aqueous solution with NaBH_4_ to yield a red colloidal sol. Subsequently, hydroquinone was added to the sol before the Na_2_PdCl_4_ aqueous solution was introduced at a constant rate. Following a complete addition of the Na_2_PdCl_4_ solution, the reaction was quenched by adding aqueous HCl, and the bimetallic nanoparticles were stabilized with PVP. The nanoparticles were deposited onto acidified TiO_2_ by adding a certain amount of the PdAu bimetal solution to TiO_2_ slurry. Finally, the solid was filtered and washed with ultrapure water, dried at 60 °C and stored. Lately, some novel Pd based bimetallic nanostructures have been fabricated through ingenious routes involving a colloidal synthesis step. For instance, Luneau et al. [116,123] reported that Pd_0.04_Au_0.96_ nanoparticles embedded in porous SiO_2_ were fabricated by a modified colloidal synthesis method, the proto-raspberry templating approach. Generally, citrate capped Au nanoparticles (~5 nm) were prepared by the reduction of HAuCl_4_ with NaBH_4_ in deionized water, followed by adding 5 mL of ascorbic acid aqueous solution (0.1 M). Next, 40 mL of the slurry was mixed with 150 μL of Pd(NO_3_)_2_ aqueous solution (10 mM) and another 5 mL of ascorbic acid aqueous solution (0.1 M), followed by stirring for 12 h at room temperature and then stored at 4 °C to obtain a Pd_0.04_Au_0.96_ nanoparticle suspension. After that, a specific amount of the suspension was mixed with a thiol-modified polystyrene colloid (PS-SH) solution, followed by agitation for 2 h. After centrifugation and washing, the obtained slurry was re-dispersed in DI water to give ~5 wt% PS@Pd_0.04_Au_0.96_ raspberry colloids. Subsequently, the PS@Pd_0.04_Au_0.96_ raspberry colloids were dried at 65 °C and then backfilled dropwise with a certain amount of prehydrolyzed TEOS solution. The slurry was washed and dried, and then calcined at 500 °C for 2 h to remove organic ligands and volatiles to obtain raspberry colloid-templated (RCT) SiO_2_ embedding PdAu bimetallic nanoparticles, Pd_0.04_Au_0.96_ RCT-SiO_2_, a catalyst with an inverse opal structure. Maligal-Ganesh et al. [115] and Liu et al. [114] designed and synthesized novel bimetallic–metal oxide spheres in an ingenious way involving colloidal synthesis steps. The schematic synthesis as well as morphology and structure characterization results involved in Refs. [114,115,116,123] are shown in Figure 11.

Nevertheless, traditional studies suggest that the ligand adversely affects catalyst performance and needs to be removed before use. Removal of the ligand without changing the structure and surface morphology of the catalysts remains a challenging task for the colloidal synthesis method. Moreover, relatively sophisticated synthetic procedures also prevent the scaling up for productive applications [59,72,124]. On the other hand, however, a growing body of complementary theoretical calculations and experimentally detailed characterization studies [38,83,118,125,126,127,128] has shown that appropriate ligand modification affected the coordination structure and spatial arrangement of the exposed active sites on the metal nanoparticle surface. This could positively impact the adsorption accessibility and adsorption modes of the reactant species on Pd atoms (geometric effects), thus improving the selectivity of ethylene in the acetylene hydrogenation reaction. Moreover, the appropriate ligand could also tune the energy landscape felt by reactive species close to the active sites (electronic effects). In some cases, the ligand may also form an active interface with metal atoms, preventing the formation of Pd hydrides as well as changing the catalytic reaction pathway (co-catalytic effects).

#### 2.1.8. Hydrothermal/Solvothermal Method

The synthesis of nanomaterials by the hydrothermal/solvothermal method is one of the most attractive fields in materials science to date. This method refers to the synthesis technique using the chemical reactions of substances in a saturated solution at a given temperature (100~1000 °C) and pressure (1~100 MPa). It provides a special physical and chemical environment for the reaction and crystallization of various precursors, which cannot be obtained under normal pressure conditions, so that some chemical reactions that are difficult or even impossible to occur under normal conditions can be carried out smoothly in an autoclave. By this method, it is conducive to the preparation of dispersed and crystalline powders, and the reaction is conducive to the synthesis of toxic systems in closed containers, reducing the pollution to the environment [129,130]. However, there are few publications about supported Pd-based bimetallic catalysts prepared by this method for selective hydrogenation of acetylene. There has been only one to date [131].

**Figure 12:**
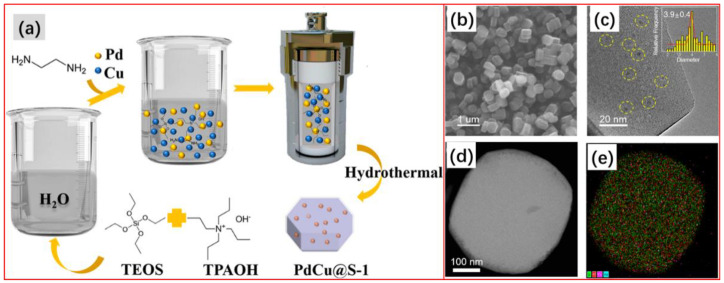
(**a**) Schematic synthesis of PdCu@S-1 by the hydrothermal method; (**b**,**c**) TEM images, as well as (**d**,**e**) elemental mapping of PdCu@S-1. (Figure reproduced with permission from Ref. [131]. Copyright 2022, ACS Publications).

The Luo group [131] reported alloyed PdCu nanoparticles within siliceous zeolite crystals, PdCu@S-1, by the hydrothermal method for selective hydrogenation of acetylene to ethylene. First, 14.88 g of tetrapropylammonium hydroxide (TPAOH (40 wt%)) was added into 90.24 g of DI water and stirred for 10 min in one flask, followed by the slow addition of 26.16 g of tetraethylorthosilicate (TEOS, >99%). After stirring at room temperature for 6 h, a clear and transparent solution was obtained. Next, 0.285 g of Cu(NO_3_)_2_·3H_2_O and 0.75 mL of Na_2_PdCl_4_ (20 mg/mL) were dissolved in 5 mL of DI water in another flask and stirred for 30 min, followed by the addition of 0.606 g of ethylenediamine and then stirred for another 30 min. After that, the mixture from the two beakers was poured together and blended at room temperature for 10 min, and then transferred into an autoclave and kept at 180 °C for 96 h. After calcination at 550 °C for 4 h in the air atmosphere, the PdCu@S-1 sample was obtained (Figure 12a). TEM characterizations confirmed the successful synthesis of ZSM-5 (MFI) zeolite with a uniform morphology and size distribution (200–500 nm) (Figure 12b); moreover, the average size of PdCu nanoparticles in PdCu@S-1 was 3.9 nm (Figure 12c). EDS mapping showed that the element distribution of palladium and copper is highly uniform and consistent (Figure 12d,e).

#### 2.1.9. Surface Inorganometallic Chemistry

**Figure 13:**
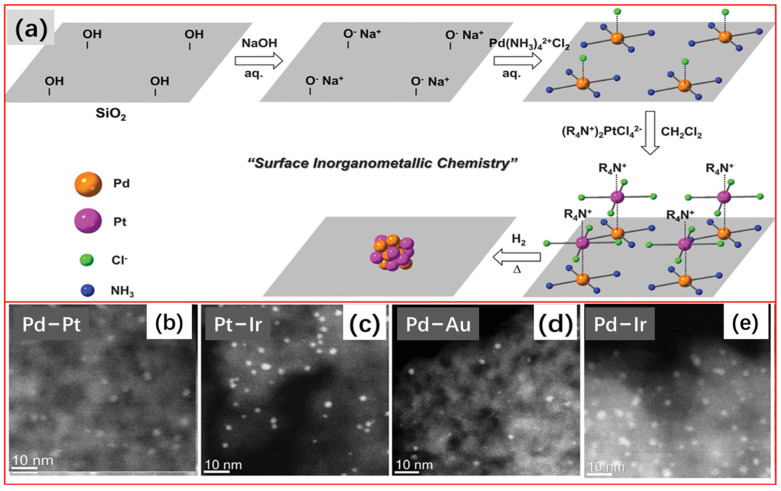
(**a**) Schematic illustration for the synthesis of supported bimetallic nanoparticles by the surface inorganometallic chemistry approach, and (**b**–**e**) HAADF-STEM images of the 10 types of supported Pd-based bimetallic nanoparticles (Figure reproduced with permission from Ref. [132]. Copyright 2018, AAAS).

Lately, the Ding group [132] proposed a novel and general strategy for the synthesis of the supported bimetallic nanoparticles called the surface inorganometallic chemistry approach. The schematic illustration is shown in Figure 13a. Taking the synthesis of the SiO_2_-supported PdPt bimetallic catalyst as an example, the synthesis process is described briefly. A porous silica support is first dispersed in water, followed by the adjustment of the pH of the solution to 9 to ensure a negatively charged SiO_2_ surface. After the adsorption of complex metal cations, Pd(NH_3_)_4_^2+^, silica is separated from the slurry, then washed and dried for the subsequent adsorption of complex metal anions. Next, PtCl_4_^2–^ anions are dissolved in an aprotic solvent, dichloromethane, with the assistance of quaternary ammonium cations, tetraoctylammonium (TOA^+^). The CH_2_Cl_2_ solution of PtCl_4_^2–^ anions are added to the SiO_2_-supported Pd(NH_3_)_4_^2+^ cations to form SiO_2_–supported double complex salts (DCSs). After centrifugation from the mother solution, washing with CH_2_Cl_2_, and then drying at room temperature for 12 h, the SiO_2_-supported Pd(NH_3_)_4_ PtCl_4_ DCSs were reduced by hydrogen at 400 °C for 30 min to obtain PdPt/SiO_2_. The as-prepared supported bimetallic nanoparticles are all ultra-small, ranging between 1 and 3 nanometers in diameter (Figure 13b). The PdPt/SiO_2_ catalyst showed enhanced catalytic performance in the selective hydrogenation of acetylene.

### 2.2. Plasma Treatment

Our group has been dedicatedly involved in the plasma-assisted preparation of meta-based catalysts for the selective semi-hydrogenation of acetylene [133,134,135,136]. In this part, combining our work, we will expound the classification and characteristics of plasma as well as its advantages in catalyst preparation and, subsequently, summarize the progress of plasma in assisting the preparation of Pd bimetallic catalysts for acetylene selective hydrogenation catalysts.

**Figure 14:**
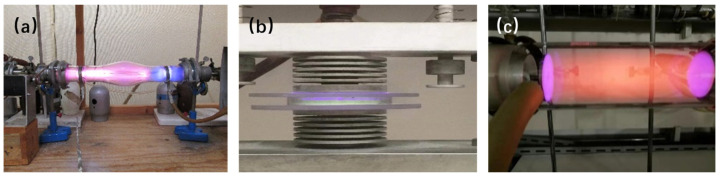
(**a**) RF, (**b**) DBD, and (**c**) glow discharge plasma generators and the corresponding gas discharge plasma phenomenon.

Plasma is a partially ionized gas consisting of molecules, ions, radicals, electrons, photons, and other excited species, also known as the fourth state of matter [137]. About 99% of the matter in the universe exists as plasma. For instance, lightning and polar lights often seen in nature are plasma. Depending on their temperature, ionic density, and energy level, plasmas are usually classified as high-temperature (equilibrium) plasmas and low-temperature (non-equilibrium) plasmas (including thermal and non-thermal plasmas). In thermal plasmas, the gas bulk temperature can reach up to 100,000 K or more. By contrast, the bulk temperature of non-thermal plasmas can be as low as room temperature, or even below. If the gas bulk temperature is close to room temperature, a non-thermal plasma is also called cold plasma. High-temperature plasmas can be created by nuclear fusion and be a clean energy the future of humanity. On the other hand, low-temperature plasmas are important for the syntheses and processing of matters, including catalyst preparation [133,137]. Plasmas used in laboratories and industry are mostly created by applying AC or DC high voltage to a gas phase. They are normally referred to as gas discharge plasmas. The cold plasmas generated with different electrode configurations or under different operating conditions are very different. Glow discharge, dielectric barrier discharge (DBD), and radio frequency (RF) discharge are three conventional cold plasma phenomena [137,138,139]. Figure 14 shows the generation setups of the three plasmas in our group, and the details have been described in our previous works [133,137,140].

There are two key characteristics of cold plasma. One is that it is a partially ionized gas containing a lot of highly active species, e.g., ions, electrons, radicals, photons, and excited species. The other is a far-from-equilibrium (non-equilibrium) state, which means that even the lighter species, such as electrons in the cold plasma, can be very “hot”, up to several thousand degrees Celsius or even more, while the bulk temperature remains as low as room temperature [133,137,138,139]. The combination of the two key characteristics enables cold plasmas for catalyst preparation in unique ways compared to conventional thermal treatment. As a result, cold plasmas regulate structures, morphologies, and the interaction between support and metal, as well as induce processes at surfaces in a more efficient, controlled, and mild way compared with the traditional thermal methods.

**Figure 15:**
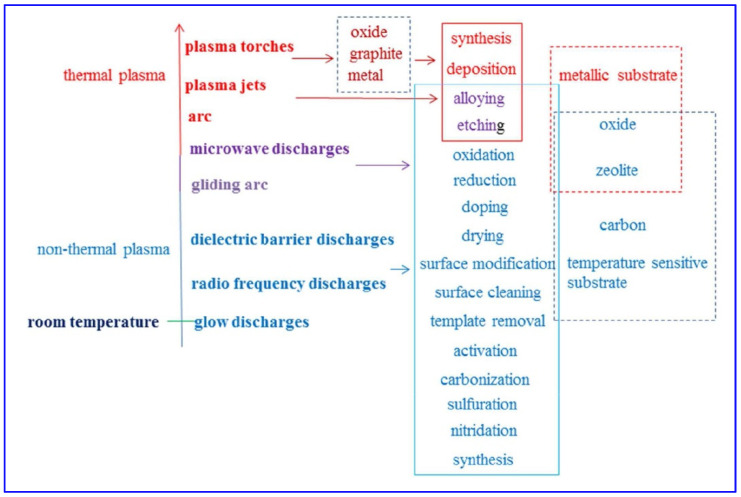
Overview of plasma types and their most common applications in catalyst preparation. (Figure reproduced with permission from Ref. [133]. Copyright 2018, ACS Publications).

Our group and other coworkers [133] have summarized common applications in catalyst preparation with some typical plasmas as shown in Figure 15. Different types of plasmas can be obtained depending on power-plasma coupling, electrode configuration, and the dielectric material used, such as glow discharges, radio frequency discharges, dielectric barrier discharges, plasma torches, etc. Some are thermal plasmas, and some are non-thermal or room temperature plasmas. All of these discharges have been applied to catalyst preparation procedures, including synthesis, etching, reduction, doping, template removal, activation, etc., to synthesize metal, oxide, zeolite, carbon, oxide, and/or temperature sensitive materials. We have prepared several Pd-based monometallic and bimetallic catalysts supported on various metal oxides by RF plasma assistance under different gas atmospheres for selective semi-hydrogenation of acetylene [86,134,140,141,142,143,144]. Plasma treatments were all performed in a custom-made 360° rotating RF plasma system [145]. Typically, procedures for RF plasma-assisted preparation of the catalyst are as follows: First, 1 g of fresh catalyst precures obtained by conventional impregnation or deposition-precipitation are dried for 12 h, and then loaded in a RF plasma chamber for plasma treatments for 30 min. The operating parameters for the RF plasma generator are as follows: 400 mTorr pressure, a continuous wave, and 160 W output. Finally, after plasma treatment, the samples are further reduced by thermal reduction in H_2_ for use.

Research showed that H_2_ plasma treatment can reduce supported Pd nitrate, PdO, PdO/Au, and PdO/AgO to Pd metals or bimetals at room temperature; in addition to the O_2_, H_2_, Ar, and air plasma treatment, they all can improve the dispersion of metal nanoparticles, and can effectively induce strong metal-support interaction. In addition, it was mentioned above that the removal of the ligand was a challenge for the colloid method. Fortunately, a cold plasma treatment can offer a possibility to effectively remove the agent. Our group [135] reported a SiO_2_-supported Au–Ag bimetallic catalyst prepared by the colloid method, using an APTES (3-aminopropyltriethoxysilane) functionalized silica gel, with HAuCl_4_ and AgNO_3_ as precursors, and NaBH_4_ as a reductant. It was found that APTES, on the Au–Ag bimetallic catalyst, could be efficiently removed by the RF-plasma treatment for 30 min at room temperature, with a continuous wave and 130 W output. In addition, cold plasma can also be used for surface cleaning or the modification of the catalyst support before metal loading. For instance, Ar plasma sputtering is generally a necessary pre-processing step of matrixes for catalyst PVD synthesis [146,147]. Moreover, Panafidin et al. [148] have reported Ar plasma sputtering on a highly oriented pyrolytic graphite (HOPG) surface for supporting Pd–In bimetals. The experiment result shows that Ar plasma sputtering effectively introduced defects which can be stabilization centers for deposition particles with a uniform distribution. Unfortunately, plasma devices normally give researchers the wrong impression of being complex and expensive and, as a result, only a limited number of groups around the world are using them to prepare catalysts. However, some of these plasma-generating devices, such as a DBD plasma generator system, are simple, easy to operate, and, especially, inexpensive. It is worth having more researchers into this field to contribute wisdom.

### 2.3. Thermal Pyrolysis

**Figure 16:**
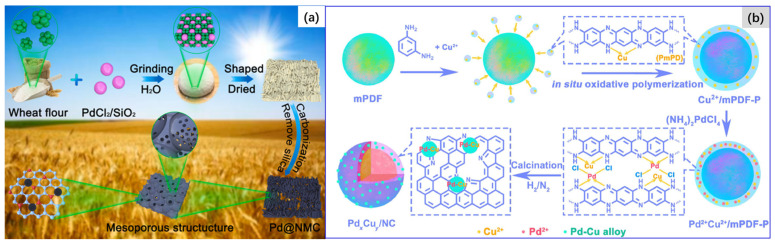
Schematic diagram for the synthesis of nitrogen-doped mesoporous carbon (NC)-supported (**a**) Pd catalyst derived from a physical mixture of Pd cations and biomass material. (Figure reproduced with permission from Ref. [57]. Copyright 2021, Elsevier B.V.), and (**b**) a PdCu bimetallic catalyst derived from the pre-prepared polymeric metal complex (Figure reproduced with permission from Ref. [149]. Copyright 2021, John Wiley and Sons, Inc.).

The thermal pyrolysis of precursors is another promising method for the preparation of carbon-supported Pd-based bimetallic catalysts. The catalyst precursors can include a physical mixture of metal cations, or a small molecular metal-organic complex as metal source and biomass material, or polymeric organics as the source of carbon support [57] (Figure 16a); moreover, it can also be polymeric metal complex formed by the coordination of the grafting of metal ions and the polymer [149] (Figure 16b).

After high temperature calcination under an inert gas atmosphere, carbon supported Pd-based bimetallic catalysts can be obtained. The keys to success in preparing samples by this method are that the screened support precursors generally need to contain N, S, P, etc., in easy coordination with metal atoms, so as to anchor and immobilize the metal particles formed during the thermal pyrolysis process, and avoid aggregation, as well as possess a specific surface area and appropriate pore structure after pyrolysis. Since the discovery of metal–organic frameworks (MOFs) and covalent organic framework (COFs) materials, the fabrication of MOFs- [150,151,152,153] and COF- [63,154,155] derived carbon supported metal nanoparticles has received more and more attention, due to their abundant intrinsic molecular metal sites, ordered pore structures, large surface areas, as well as chemical tunability. MOF- and COF-derived carbon materials have a porous structure and a high surface area, as well as abundant nitrogen atoms, which can anchor metal atoms. Our group [153] reported a series of carbon nanosheet-supported Pd–Zn inter-metallics (Pd–Zn-ins/CNS) prepared by a one-step thermal pyrolysis method with new palladium–zinc MOFs for selective hydrogenation of acetylene under simulated front-end conditions. Sarkar et al. [156] impregnated a methanol solution of palladium nitrate into Cu-BTC-MOFs (HKUST-1) as the catalyst precursor, and then carbonized it at 450 °C under N_2_ atmosphere for 3 h to obtain the targeted catalyst. This method is simple, using existing mono-metallic MOFs to obtain a Pd-based bimetallic catalyst precursor by impregnation with a Pd metal salt. However, under this mode, the structure, morphology, and especially the particle size of catalysts is not easy to control. Hu and coworkers [150] first obtained carbon-supported ZnO material by carbonizing ZIF-8 MOFs, which were then impregnated with a Na_2_PdCl_4_ solution. After that, the resulted precipitates were collected, washed with methanol, and then dried in a vacuum at 50 °C overnight. After further reduction by hydrogen at 400 °C, the targeted catalyst was obtained. The average particle size of the catalyst is only ~2 nm. Thermal pyrolysis of MOFs can achieve high metal loading while maintaining excellent dispersion and a small particle size. Especially, it is eminently suitable for the fabrication of various single atom or single atom alloy catalysts [63,155,157,158]. However, high preparation costs are an unavoidable issue for this method.

### 2.4. Vapor Deposition and Electrochemical Deposition

**Figure 17:**
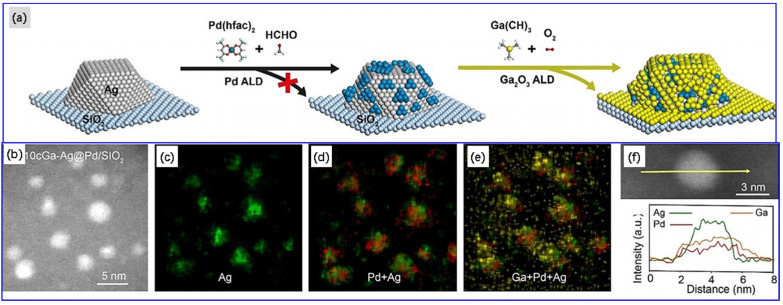
(**a**) Schematic illustration of the ALD synthesis of Ga_2_O_3_-coated Pd@Ag/SiO_2_ catalysts; (**b**) STEM image of Ga_2_O_3_-coated Ga–Ag@Pd/SiO_2_ and the corresponding EDS mapping of (**c**) Ag, (**d**) Pd + Ag, and (**e**) Ga + Pd + Ag; (**f**) STEM image of a Ga_2_O_3_-coated Pd@Ag nanoparticle, along with EDS lines of Ag, Ga, and Pd (Figure reproduced with permission from Ref. [160]. Copyright 2021, John Wiley and Sons, Inc.).

Although vapor deposition is a time-honored technique and can be traced back to centuries ago, it is still one of the most important methods for sample preparation in surface science and is mainly used for the preparation of bimetallic catalysts, especially single atom alloy model catalysts in catalysis area. At present, physical vapor deposition (PVD) and atomic layer deposition (ALD) are the two main types of used methods for vapor deposition. The PVD route [69,93,159] is linked to the physical vaporization, such as metal or metal alloys at the atomic level, and deposition of a metal overlayer on a secondary metal substrate in the form of an atomic film, applying suitable parameters, such as the rate of metal vapor generation, time of deposition, substrate temperature, and gas atmosphere or ultra-high vacuum, and a suitable environment, such as in a vacuum or in a selected gas. For instance, Zimmermann et al. [159] utilized the PVD method to prepare single-phase and catalytically active GaPd_2_ coatings deposited on borosilicate glass, Si(111), and planar, as well as micro-structured stainless steel foils, respectively. EDX measurements showed a homogeneous distribution of Ga and Pd in the thin film. XRD results indicated the formation of intermetallic GaPd_2_. ALD is a method in which gas precursor molecules are pulsed alternately into an ALD reactor and chemically adsorbed on the solid substrate surface and occur the ordered self-terminated surface reaction to form metallically atomic layers [90,93]. The ALD method has been successfully used in the synthesis of supported bimetallic NPs with control in metal particle size, morphology, and composition [93,160]. Liu et al. [160] reported that by using the ALD method, a Ga_2_O_3_ coating with an Ag@Pd core–shell bimetallic nanoparticle catalyst was synthesized for the selective hydrogenation of acetylene. HAADF-STEM results verified the presence of Pd shell-forming islands on Ag NPs with a thickness of 1–2 atomic layers. The average particle size of the Ga_2_O_3_-coated Pd@Ag/SiO_2_ nanoparticle is only ~4 nm. The schematic illustration of the synthesis of the Ga_2_O_3_-coated Pd@Ag/SiO_2_ catalyst and its HAADF-STEM images and EDS line profiles are shown in Figure 17. ALD is also increasingly being used to prepare SAA catalysts [90,93].

Electrochemical deposition (ECD) refers to the process in which given raw materials are dissolved in solution, under external power supply, through oxidation reaction on the anode of the electrolytic cell or ion reduction reaction on the cathode, as well as electro-crystallization, forming metallically atomic layers on the surface of the solid substrate [8]. The Bu group [8] has synthesized Cu dendrites for the selective electrocatalytic semi-hydrogenation of acetylene impurities, exhibiting a high specific selectivity of 97%. As far as we know, supported Pd-based bimetallic catalysts prepared by the ECD method for hydrogenation of acetylene have not been reported so far. It is also an interesting approach for obtaining bimetallic catalysts such as ECD and PVD, owing to the fact that the particle size, morphology, and composition of the coated metal may be controlled by researchers.

### 2.5. Ball Milling

Ball milling is a method that has been gaining momentum recently and is suitable for the preparation of supported catalysts most recently [2,69]. A pulverisette planetary micro mill and milling equipment made of zirconia, including a milling jar, and grinding balls of different sizes, are the key devices for this method. Using this method, several α-Al_2_O_3_-supported Pd–Ag bimetallic catalysts were prepared by the Kley group [2]. Typically, the coarse metal powders (about 5 wt% of Pd and Ag) and support precursor (boehmite, γ-AlOOH) were loaded in one pot and ball-milled to obtain a concentrated batch. Thereafter, under suitable ball-milling conditions, boehmite dehydrates to form α-Al_2_O_3_ with a remarkably high specific surface area, concomitantly Pd–Ag alloy nanoparticles formed on the resulting HSA-α-Al_2_O_3_ to obtain the final catalyst. Determining the optimization of the reaction conditions is the key to success for the method. Experimental results show that catalysts prepared via ball milling possess characteristics of smaller particle size, more uniform distribution, and better stability compared to those prepared by the conventional impregnation method.

## 3. Synthesis of Single Atom Alloy and Bimetallic Dual Atom Catalysts

Since the concept of single-atom alloys (SAAs) was proposed, in 2012, by Sykes’ group [146], it has rapidly become a new research frontier in the field of materials science and catalysis, both theoretically and experimentally [93]. In general, SAAs is a class of bimetallic complexes consisting of a metal matrix and active metal atoms dispersed on the matrix in the form of individual atoms. Active metal atoms are held in place by the interactions with the metal atoms of the matrix that surrounds them without forming metallic bonds between each other. Different from traditional alloys, the special geometric structures have result in the unique geometric structures, as well as the synergistic effects of SAAs. All these characteristics contribute to the extremely high atom utilization and correspondingly reduce the use of noble metals. On the other hand, effective modulation of the adsorption strength and activation energy, tuning the reaction pathway, as well as breaking the Brønsted–Evans–Polanyi (BEP) relationship consequently offer an excellent catalytic activity and selectivity [69,90,93,161,162]. According to the adsorption mode of the reaction intermediates in acetylene hydrogenation mentioned above, isolated Pd atoms are favorable for the formation of single site adsorption structures with reaction intermediate species further desorbing to ethylene and not ethane, and consequently improving ethylene selectivity for the hydrogenation of acetylene. In this context, Pd_1_ SAAs are undoubtedly the most potential catalysts for the selective hydrogenation of acetylene. It is worth mentioning that the first report of SAAs is coincidentally in preparation, including its performance for the selective hydrogenation of acetylene by Sykes’ group [146]. Since then, many supported Pd_1_ SAAs have been reported for the selective semi-hydrogenation of acetylene.

Despite supported SAAs, it is promising to minimize the usage of noble metals, but the noble active metal loadings are relatively low, due to the need of guaranteeing that the active metal atoms are distributed as single atoms in the secondary metal matrix, often resulting in a relatively low overall activity. Theoretically, under the conditions of maintaining a certain amount of SAAs loading, further reducing the particle size is conducive to exposing more active sites. With that in mind, most recently, dual-atom catalysts (DACs) have attracted considerable research interest [163]. Just as the name implies, bimetallic DACs are a kind of catalyst that requires the introduction of another atom to combine the parent active metal, forming dual active sites in isolation; it can be viewed as SAAs catalysts for particle size reduction to the limit case. By reason of unique electronic, adsorption and synergistic effects, bimetallic DACs are also a very suitable catalyst system for the selective hydrogenation of acetylene [41,163,164,165,166].

**Figure 18:**
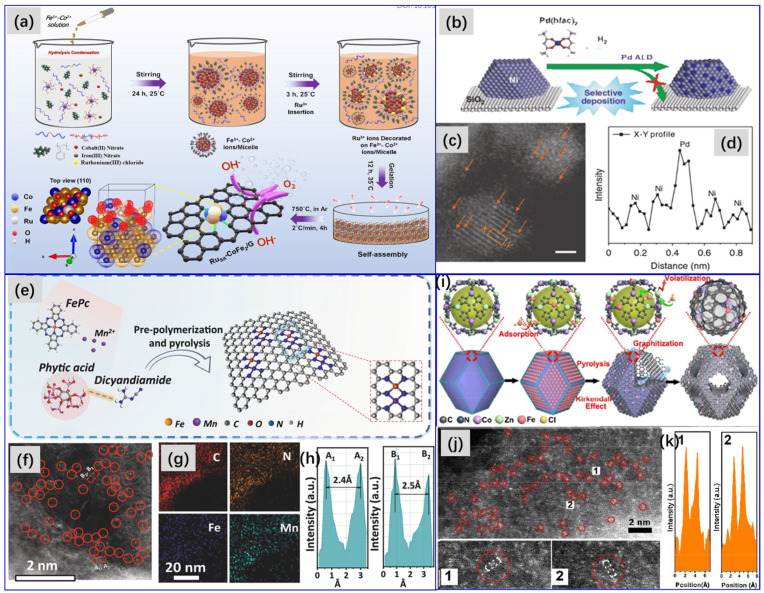
(**a**) Schematic synthesis process of the graphitic-carbon encapsulated Ru single-atom on the cobalt–iron bimetallic-alloy, Ru_SA_CoFe_2_/G, with the micelle-incorporated sol-gel method, followed by carbothermal reduction. (Figure reproduced with permission from Ref. [174]. Copyright 2020, Royal Society of Chemistry). (**b**) Schematic illustration of the synthesis of a SiO_2_-supported Pd single-atom on Ni nanoparticles, using selective Pd ALD; (**c**) HAADF-STEM image of 5PdNi/SiO_2_ obtained by five cycles of Pd ALD. The Pd single atoms on the Ni NPs are highlighted by brown arrows. (**d**) Intensity profile along the line X-Y, (**c**) further confirming the presence of Pd single atoms (Figure reproduced with permission from Ref. [178]. Copyright 2019, Nature Publishing Group). (**e**) Schematic illustration of the synthesis procedure for NC-supported (Fe,Mn) bimetallic dual-atom catalysts, (Fe,Mn)/NC. (**f**) HAADF-STEM image of (Fe,Mn)/NC, and some of the (Fe,Mn) dual-metal sites are highlighted by red circles. (**g**) HAADF-STEM image of Fe,Mn/NC with EDS mapping. (**h**) The intensity profiles of the two bimetallic Fe–Mn sites (Figure reproduced with permission from Ref. [180]. Copyright 2021, Nature Publishing Group). (**i**) Schematic illustration for the preparation of NC-supported (Fe,Co) bimetallic dual-atom catalysts, (Fe,Co)/N-C. (**j**) HAADF-STEM images of (Fe,Co)/NC, and some of the Fe–Co dual sites were highlighted by red circles. (**k**) Intensity profiles obtained on the two circled Fe–Co dual sites in (**j**) 1 and 2 (Figure reproduced with permission from Ref. [179]. Copyright 2017, ACS Publications).

In the case of supported Pd_1_ SAAs catalysts, the preparation methods vary, and the technical process has been relatively mature, although most of the methods for the preparation of bimetallic nano catalysts are still suitable and have been optimized and employed for their synthesis, such as incipient wetness impregnation (IWI) [18,167,168], deposition-precipitation (DP) [169], galvanic replacement (GR) [170,171], controlled surface reaction (CSR) [97], colloidal electroless reduction deposition (colloidal eless-RD) [172,173], the sol-gel method [174], physical vapor deposition (PVD) [146,147,175], electrochemical deposition (ECD) [176], photochemical reduction (PR) [177], and atomic layer deposition (ALD) [178]. For supported bimetallic DACs, the currently reported synthesis methods include photochemical reduction (PR) [164], deposition-precipitation (DP) [41], as well as thermal pyrolysis and even subsequent acid leading [179,180]. The illustrations for the specific synthetic methods, as well as characterization results of SAAs and DACs are shown in Figure 18.

## 4. Conclusions

1. In summary, the synthesis of supported Pd-based bimetallic catalysts for selective semi-hydrogenation of acetylene is possible by a variety of fabrication procedures. However, it also needs to be aware that each synthesis method has its own characteristics, whether it is old or new; different methods have different application conditions and may be applicable to different catalyst systems. In order to establish a clear comparison of different synthesis strategies, their key characteristics, advantages, and disadvantages, and the catalyst systems that they have been applied to, are listed in Table 1.

2. The continuous improvement of the methods and their use in appropriate combinations will continue to be a research focus, contributing to the further improvement of the palladium-based bimetallic catalyst performance.
3. In any case, however, the ultimate application of palladium-based bimetallic catalysts for the selective semi-hydrogenation of acetylene is an industrial-scale reaction scenario. Therefore, to develop more simple and efficient catalyst preparation methods to meet the requirements of industrial-scale reactions will be one of the goals that researchers are constantly striving for.
4. The development of preparation methods with the participation of environmentally benign visible or ultraviolet (UV) light, cold plasma, and other new energy sources should be a development direction.
5. Dual-atom catalysts (DACs) theoretically can achieve the same high loadings as nano-catalysts and, at the same time, can achieve the same mono-dispersion of active metal atoms as single-atom alloy catalysts. With that in mind, supported Pd-based dual-atom catalysts should be the most promising alternative for selective semi-hydrogenation of acetylene in the future.
6. Most recently, machine learning (ML), combining experiments, has brought new solutions for the screening of active metals and suitable supports and for the performance optimization of catalyst systems [43,185]. The Liu group [43,185] performed pioneering works for the design of supported PdAg bimetallic catalysts for the acetylene semi-hydrogenation reaction. Reaction test results indicated a new record for the low-temperature selective semi-hydrogenation of acetylene: 97.2% selectivity and 100% acetylene conversion below 100 °C; moreover, the resulting Pd_1_Ag_3_/r-TiO_2_ is rather stable, above 95% selectivity at the high conversion (>98%) over a 120 h experiment. Thus, it could be reasonable to speculate that emerging science such as artificial intelligence (AI), machine learning (ML), big data, and supercomputing combined experiments demonstrate a broad prospect for the rational and efficient design of complex heterogeneous catalytic systems.

## Figures and Tables

**Table 1:** Summary for the preparation methods [69,73,87,92,93,95,181,182,183,184].

Synthetic Method	Key Features	Synthesized Catalyst Types	Merits	Demerits	Example and Ref.
Incipient wetness impregnation (IWI) method	Most widely used preparation method	Supported bimetallic nano-catalysts/single atom alloy	Easy operation; low cost; high production capacity; sample resistance to sintering; SMSI	Minimal control of particle size and element distribution; calcination contamination	Pd_1_Ag_3_/r-TiO_2_ (T750) [185]
Co-precipitation (CP)	Simultaneous precipitation of the two metals and the support	Supported bimetallic nano-catalysts	Easy control of particle size and element distribution; maximum metal loading and metal-support interaction	Accurate control of synthesis conditions; impurity (by precipitant) removal is required; small proportion of active components on the surface; thermal treatment	Mco-PdCu/MgAl-cHT [75]
Deposition-precipitation (DP)	Widely used in the preparation of supported metal catalysts; one-pot synthesis	Supported bimetallic nano-catalysts/single atom alloy/bimetallic dual atom catalysts	Particle size and component distribution are generally superior to those prepared by IWI; SMSI	Accurate control of synthesis conditions; impurity removal is required; metal-support interaction is required; thermal treatment; easy liquid-phase nucleation	Pd_1_Cu_1_/ND@G (DACs) [41]
One-pot reduction deposition (one-pot RD)	One-pot synthesis; simultaneous reduction of the two metals to deposit on the support; chemical reducing agents participate in the reduction.	Supported bimetallic nano-catalysts	Particle size and component distribution are generally superior to those prepared by DP	Accurate control of synthesis conditions; impurity removal is required; metal-support interaction is required; thermal treatment; existing liquid-phase nucleation and mono-metal deposition	PdBi/Calcite [81]
Electroless reduction deposition (eless-RD)	Parent metal activates chemical reducing agents to reduce the secondary metal; atom-by-atom deposition on the parent metal	Supported bimetallic nano-catalysts	Available complex structures, e.g., core–shell structures; achieving deposition of the dopant metal that cannot be performed by GR	No deposition on the support is still a question to consider; in most of cases, more precise control of synthesis parameters than GR	Cu–Pd/TiO_2_ [99]
Galvanic replacement (GR)	Supported parent metal is prepared first; atom-by-atom metal exchange driven by reduction potential.	Supported bimetallic nano–catalysts/single atom alloy	Available special compositions and structures; shape of the template maintained	Complicated steps; deposition depends on the difference in reduction potentials between the two metals	Pd–Ag/SiO_2_ [101]
Controlled surface reaction (CSR)	Reduction of the parent metal occurs first; strict operation conditions	Supported bimetallic nano-catalysts/single atom alloy	Available complex structures	High-level complex steps; need to perform in an inert atmosphere and anhydrous solutions	CuPd_0.02_/TiO_2_ [97]
Photochemical reduction (PR)	Greenness; visible or ultraviolet (UV) light drives reactions	Supported bimetallic nano-catalysts/single atom alloy/bimetallic dual atom catalysts	Simplicity; high efficiency; greenness; normally room temperature and atmospheric pressure	Some metal atoms do not sensitively respond to irradiation	Pd_9_Au_1_/ZnTi [112]
Colloidal synthesis	Metal precursors form a metallic colloid to support on the matrix	Supported bimetallic nano-catalysts/single atom alloy	Easy and flexible control of particle shape, size, and element distribution	Precise control of synthesis conditions and impurity removal is generally required	Au@Pd/TiO_2_ [119]
Sol-gel method	The precursors undergo the processes of hydrolysis and polymerization, forming sol and then gel	Supported bimetallic nano-catalysts/single atom alloy	Easy control of particle size and element distribution at the atomic level; facile synthesis condition	High price of metal precursors; environmental health issues; long-time synthesis.	CuPd/ZIF-8 [79]
Physical vapor deposition (PVD)	Physical vaporization of metals; model catalyst preparation; layer-by-layer deposition on the matrix	Supported bimetallic nano-catalysts/single atom alloy	Simple steps; environmental friendliness; diverse coating material availability; controllable size and dispersion	High equipment cost; high process temperature; high surface cleanliness; UHV conditions; not suitable for deposition on a complex surface	GaPd_2_/Si(111) [159]
Atomic layer deposition (ALD)	Atomic layer-by-layer deposition; ordered self-terminated reduction occurs on the substrate surface; gaseous metal molecule precursor	Supported bimetallic nano-catalysts/single atom alloy	Atomically distributed; flexible control of the surface, composition, overlayers, and even atomic level thickness; extraordinary reproducibility	High equipment cost; high price of metal precursors; UHV conditions; slow ALD progression	Ga_2_O_3_-coated Pd@Ag/SiO_2_ [160]
Electrochemical deposition (ECD)	Reactions are driven by an outside current basis	Supported bimetallic nano-catalysts/single atom alloy	Near ambient conditions; efficient usedness of precursors; easy fabrication with excellent control of the morphology and composition with high reproducibility; inexpensive method	Need a sophisticated instrument; poor scalability	Cu dendrites [8]
Cold plasmas treatment	A partially ionized gas containing many highly active species; non-equilibrium state	Supported bimetallic nano-catalysts	A more efficient, controlled, and mild method, compared with the traditional thermal methods	Specialized equipment	Au–Ag/SiO_2_ [135]
Thermal pyrolysis	Thermal pyrolysis and carbonization of organic or polymer materials and metals with van der Waals force or chemical bonding force	Supported bimetallic nano-catalysts/single atom alloy/bimetallic dual atom catalysts	Simplicity; simultaneous one-step preparation of support and active metal	Long reaction time; not easy to control particle size and element distribution; excessive energy usage; air contamination	Pd–Zn-ins/CNS [153]
Ball milling	The simplest and most efficient mechanical process	Supported bimetallic nano-catalysts/single atom alloy	Extensive range of applications; simplicity; safety	Specialized heavy equipment is required; big power loss; contamination and noise	Pd–Ag/α-Al_2_O_3_ [2]

## Data Availability

Not applicable.
